# Strategic intelligence and organizational ambidexterity for strengthening health system performance in a public healthcare network

**DOI:** 10.1016/j.dialog.2026.100356

**Published:** 2026-10-07

**Authors:** Mohammad Khalilzadeh, Zahra Rajaei, Sayyid Ali Banihashemi

**Affiliations:** a CENTRUM Católica Graduate Business School, Pontificia Universidad Católica del Perú, Lima, Peru; b Department of Public Administration, Payame Noor University, Tehran, Iran; c Department of Industrial Engineering, Payame Noor University, Tehran, Iran

**Keywords:** Strategic intelligence, Organizational ambidexterity, Exploratory innovation, Exploitative innovation, Cross-sectional study, Structural equation modeling

## Abstract

This study examines the association between strategic intelligence (SI) and organizational ambidexterity (OA) in a public healthcare network, with attention to the two dimensions of OA: exploratory innovation (innovation, experimentation, and adaptation) and exploitative innovation (refinement, efficiency, and execution of existing competencies). OA was modeled as a latent variable indicated by the exploratory and exploitative innovation dimension scores. A cross-sectional correlational study was conducted in a public healthcare network in Iran (312 employees; 172 respondents, with the sample size guided by Cochran's formula). Persian-language versions of established self-report questionnaires measured SI (foresight, systemic thinking, vision, motivation, partnering) and OA (exploratory/exploitative innovation). Data were analyzed using structural equation modeling (SEM) in AMOS and SPSS. Strategic intelligence was positively associated with organizational ambidexterity (standardized β = 0.580, R^2^ = 0.336), exploratory innovation (β = 0.585, R^2^ = 0.342), and exploitative innovation (β = 0.639, R^2^ = 0.408). Among the SI dimensions, foresight, vision, and partnering were positively associated with organizational ambidexterity, whereas systemic thinking and motivation were not statistically significant. The associations concern employees' perceptions of organizational capabilities. No patient-level outcomes, service-quality indicators, access measures, or health-system performance data were collected; any relevance to health-system performance or Sustainable Development Goal 3 is therefore a hypothesized pathway for future research, not a result of this study. Because of the cross-sectional self-report design, the single-site sample, and the modest sample size, the findings are preliminary and should not be interpreted causally or generalized.

## 1 Introduction

Organizations must adapt to ongoing internal and external changes to ensure their survival. In light of the high demand for services and the rapidly changing environment, the focus is no longer solely on the provision of services and how they are delivered. Instead, greater importance is placed on implementing appropriate measures and tools aligned with organizational goals, human resources, and performance objectives, such as achieving organizational missions and visions [1]. Research indicates that successful organizations tend to balance managing the demands of today's operating environment while simultaneously adapting to future changes [2].

This ability, referred to as organizational ambidexterity, was first introduced by Duncan in 1976 and later developed by March in 1991, who introduced the concepts of exploitation and exploration. Researchers view exploration and exploitation as fundamentally distinct activities that organizations must pursue simultaneously to sustain performance and long-term viability [3]. According to the consensus in the literature, an ambidextrous organization is capable of both exploiting existing competencies and exploring new opportunities, thereby enhancing efficiency and adaptability [4]. This dual capability is recognized as a key factor for sustained organizational performance [4], [5].

The application of the concept of ambidexterity in the literature does not refer to managerial activities per se but rather reflects managerial capabilities [6], recognized as a dynamic capability [7]. Organizational ambidexterity, in its general sense, refers to the skill and ability of an individual to use both hands equally. In organizational literature, it signifies the capacity of an organization to perform two different tasks simultaneously [8]. In summary, organizational ambidexterity is the ability of an organization to execute critical and strategic activities effectively. Therefore, this concept is attributed to organizations that aim to avoid the trap of success and failure, which is closely tied to how exploration and exploitation are managed within the organization [7].

In other words, the goal of organizational ambidexterity is for the organization to simultaneously pursue exploration and exploitation to achieve greater flexibility and efficiency. The general consensus in the literature on ambidexterity is that an ambidextrous organization excels in both exploiting existing competencies and discovering new ones [9], [10]. Achieving ambidexterity enables organizations to enhance their performance and adaptability. However, there remains considerable ambiguity regarding the conceptualization of ambidexterity [4].

When March introduced the concepts of exploration and exploitation in 1991, he suggested that these two activities should be viewed as two ends of a continuum. In this perspective, the interaction between exploration and exploitation is seen as inevitable, and organizational ambidexterity involves managing this interaction to strike an appropriate balance between the two. However, Gupta et al. [11] considered exploration and exploitation as two independent activities. From this viewpoint, ambidexterity emphasizes an organization's capacity to simultaneously pursue the highest levels of both exploitation and exploration. Based on this, researchers have differentiated the construct of ambidexterity into two dimensions: balance and combination.

An organization that becomes ambidextrous by establishing exploration and exploitation seeks to maintain its current position while achieving a desirable future state, envisioning sustained performance. The ability to utilize the present state (exploitation) and capitalize on opportunities for future growth and survival (exploration) are the two wings propelling organizations toward ambidexterity. However, for exploration and exploitation to be considered complementary tools, their relationship must be examined and analyzed. One critical dimension in this regard, which could play a constructive role, is the role of strategic intelligence [12].

Strategic intelligence can be defined as what an organization needs to know about its operating environment to gain a clear and accurate understanding of its current interactions, anticipate and manage future changes, and adopt appropriate strategies to create value for the people it serves in both current and future contexts (definition adapted from [13] to the health-service context). Strategic intelligence typically supports senior managers responsible for designing and implementing the organization's overall strategies by providing valuable information and knowledge for strategic decision-making. It collects, networks, combines, and analyzes information from the organization's internal and external environments, assisting senior managers in gathering, analyzing, and distributing data [14].

Strategic intelligence is essentially a system comprising five key components: foresight, systemic thinking, vision formulation, motivation, and partnering [15], [16], [17], [18]. In Maccoby's [19] original formulation the fifth element is partnering, that is, the ability to build strategic alliances and productive collaborative relationships with individuals, groups, and organizations. In the Persian-language questionnaire used here (items 21–25) this dimension is labeled “collaboration”; to avoid ambiguity, it is referred to consistently as partnering throughout this article. These components enable a manager to comprehend and realize the organization's future [19]. According to McDowell [20], strategic intelligence is “the capacity for reasoning, cognition, self-awareness, learning, emotional knowledge, reasoning, planning, creativity, critical thinking, and problem-solving. It encompasses the ability to acquire, analyze, store, and retrieve information as knowledge to adapt behavior to the environment and context at the right time.”

Over the past 40 years, since strategic intelligence emerged as a new concept in research, it has often been used interchangeably with related terms such as environmental scanning and business intelligence. Researchers view strategic intelligence as a method employed for intelligent activities within the framework of strategic management and strategic planning [15], [17]. From this perspective, strategic intelligence addresses the needs of senior decision-makers in organizations and focuses on the activities of environmental scanning professionals [21].

Several studies have examined the concept of organizational ambidexterity, which refers to an organization's ability to simultaneously balance exploration and exploitation activities. Foglia et al. [22], in their article “What drives hospital wards' ambidexterity: Insights on the determinants of exploration and exploitation,” demonstrated that achieving organizational ambidexterity in hospital wards requires attention to various factors influencing the balance between exploration and exploitation, which can enhance healthcare service quality. Gutberg and Berta [23], in their study “Understanding middle managers' influence in implementing patient safety culture,” examined the role of middle managers in implementing patient safety culture and argued that these managers, as key intermediaries, must manage conflicts between operational goals and innovation to realize organizational ambidexterity in patient safety. From a strategic management perspective, Smith [24], in the article “Dynamic decision making: a model of senior leaders managing strategic paradoxes,” proposed a model enabling senior leaders to manage strategic paradoxes dynamically and maintain organizational ambidexterity.

Papachroni et al. [25], in the article “Organizational Ambidexterity Through the Lens of Paradox Theory: Building a Novel Research Agenda,” analyzed organizational ambidexterity through paradox theory and proposed a novel framework for future research in this area.

Gibson and Birkinshaw [26], in the article “The antecedents, consequences, and mediating role of organizational ambidexterity,” investigated the antecedents, outcomes, and mediating role of organizational ambidexterity and reported that this capability is associated with improved performance and innovation.

Furthermore, in recent years, as organizational environments—especially in the healthcare sector—have grown increasingly complex, researchers have paid growing attention to the role of innovation strategies and organizational ambidexterity in navigating VUCA conditions [27]. In this context, several key studies have explored these dynamics across various sectors, offering a solid theoretical foundation for this research.

Rodrigues [28] argues that while public-private partnerships (PPPs) aim to improve efficiency and service quality, they often face structural issues such as lack of transparency, conflicting interests, and weak performance monitoring. Similarly, Basabih et al. [29] highlight that although PPPs can expand service capacity, they also pose risks like increased inequality, rising healthcare costs, and tension between social and commercial goals—underscoring the need for better regulatory frameworks. Similarly, Jonassen et al. [30] explore the emotional and design-related paradoxes in remote patient monitoring technologies. Their findings suggest that embracing a paradox mindset can help healthcare professionals and designers balance technical efficiency with human-centered care, promoting a form of organizational ambidexterity that is crucial in digital healthcare environments. Eld & Johansson [31] argue that in VUCA contexts, organizations need flexible, experimental, and rapid innovation strategies, supported by continuous learning and decentralized decision-making. Suryosukmono et al. [32] explore the impact of change on public sector performance in the VUCA era, highlighting the need for adaptability, transparency, and responsive systems. Fernández-Pérez de la Lastra & Sánchez-Gardey [33] reconceptualize organizational ambidexterity for international VUCA settings and propose a research agenda focused on balancing exploration and exploitation globally. Papachroni et al. [25] use paradox theory to explain how organizations can embrace internal tensions between innovation and stability to build sustainable strategies in uncertain environments. The reviewed literature provides a valuable foundation for examining how strategic intelligence may be related to organizational ambidexterity in the healthcare sector. Specifically, these studies highlight the importance of adaptive innovation strategies and ambidextrous decision-making in navigating VUCA conditions—insights that are crucial for healthcare organizations operating in increasingly dynamic environments.

Strategic intelligence and organizational ambidexterity are conceptually complementary capabilities that may help organizations cope with complex environments. Conceptually, strategic intelligence provides analyzed information that can inform strategic decisions and may support organizational ambidexterity, allowing organizations to pursue current performance and new opportunities at the same time. This proposition is examined empirically in the present study as an association, not as a demonstrated effect.

The healthcare industry is facing significant changes and transformations [34]. Healthcare organizations must adapt to these changes to ensure their survival. Healthcare organizations have a responsibility to deliver safe, equitable, and high-quality services. Organizational capabilities such as strategic intelligence and ambidexterity have been discussed as potential enabling conditions for how healthcare institutions respond to changing service demands and public health emergencies [35]. Whether these capabilities are related to measurable health-system outcomes, however, is an empirical question that the present study does not address. Any link to Sustainable Development Goal 3 (Good Health and Well-being), including Target 3.8, is therefore treated in this article as a hypothesized, indirect pathway for future research and not as a finding. Managers of healthcare organizations should foster a culture open to positive changes to improve performance. Establishing such a culture requires an appropriate plan and strategic intelligence, which helps healthcare managers effectively utilize limited resources. Given the importance of healthcare organizations' mission, this study attempts to bridge the research gap in the relationship between strategic intelligence and organizational ambidexterity. The results may inform future research in comparable healthcare organizations.

Senior managers should focus on entrepreneurial actions to solve problems, find innovative solutions, and engage employees. Discovering new opportunities while simultaneously utilizing existing capacities and capabilities, or achieving ambidexterity, is critical in this context.

This research is both essential and innovative because it addresses a critical gap in the literature and responds to the dynamic and complex challenges faced by healthcare organizations in VUCA environments. On the one hand, the distinctive management structures, resources, and objectives of public and private sectors have created a complex and multilayered environment within the healthcare system [36]. To further ground the study's broader mechanisms in recent empirical healthcare research, complementary evidence from healthcare settings on organizational learning [37], [38], participatory and supportive organizational environments [39], workforce adaptability and collaborative competencies [40], and resource and work-environment management [41] is noted here; none of these studies examined strategic intelligence or organizational ambidexterity directly, but they support the plausibility of the organizational learning, participation, adaptability, collaboration, and resource-management mechanisms discussed throughout this article. In such a context, organizations operating in this field are compelled to pursue both the exploitation of existing structures and resources, and the exploration of innovative and emerging technologies such as telemedicine, big data analytics, and advanced therapies like gene therapy. The rapid advancement of technologies, such as the emergence of artificial intelligence, telehealth, and technology-based treatments, requires healthcare organizations to continuously adapt. Additionally, the multiplicity of stakeholders, including patients, physicians, insurers, regulators, and other health-system actors, adds to the complexity of managerial decision-making. On the other hand, crises such as the COVID-19 pandemic and the emergence of novel diseases have rendered the future increasingly uncertain and unpredictable. The COVID-19 pandemic exemplified the volatility and complexity (VUCA) that healthcare systems must navigate, but such disruptions persist through evolving challenges like digital transformation, demographic shifts, and resource scarcity [31]. Thus, SI may remain relevant to organizational ambidexterity (OA) beyond specific crises, as organizations adapt to continuous uncertainty. In such a volatile, uncertain, complex, and ambiguous (VUCA) environment, strategic intelligence capabilities may be associated with organizational ambidexterity. Although previous studies have investigated strategic intelligence and organizational ambidexterity from management and organizational perspectives, limited empirical research has examined these capabilities within public healthcare organizations. Addressing this gap is worthwhile because managerial capabilities have been argued to shape healthcare organizations' capacity to deliver safe, efficient, and patient-centered services [35], although that link is not tested here.

Despite growing interest in organizational ambidexterity (OA), three critical gaps persist in healthcare research. First, while OA is well studied in the private sector, its application in healthcare, a sector with unique structural and regulatory constraints, remains underexplored. Second, strategic intelligence (SI) is often examined in isolation from OA, with limited empirical evidence on how SI dimensions (e.g., foresight, partnering) differentially relate to exploratory vs. exploitative innovation. Third, prior studies rely heavily on qualitative or single-case designs [22], lacking structured quantitative models to test these relationships. This study addresses these gaps by proposing a SEM-based framework to analyze SI-OA linkages in healthcare, offering context-specific empirical insights into the relationship between strategic intelligence and organizational ambidexterity within a public healthcare organization. The proposed framework may serve as a foundation for future studies in comparable healthcare settings, although its broader applicability requires empirical validation in different organizational and healthcare system contexts.

This study contributes by: (1) Decomposing SI into five empirically testable dimensions (foresight, systemic thinking, vision, motivation, partnering) to identify their distinct roles in OA; (2) Employing SEM to examine the association of SI with exploratory and exploitative innovation—a approach rarely applied in healthcare settings; and (3) Contextualizing findings within post-pandemic VUCA challenges, in which organizational adaptability is of particular interest [33].

In this study, strategic intelligence (SI) is treated as the explanatory (predictor) variable and organizational ambidexterity (OA) as the outcome variable. OA is represented by two dimensions: exploratory innovation (the capacity for experimentation, new knowledge generation, and venturing into new domains) and exploitative innovation (the capacity for refining existing capabilities, improving efficiency, and executing current competencies). Three separate structural models were estimated (Fig. 1). In Model 1, OA is a latent variable indicated by the exploratory- and exploitative-innovation dimension scores, and SI is a latent variable indicated by the five SI dimension scores (H1). In Model 2, exploratory and exploitative innovation are separate latent variables, each measured by its own scale items, with SI as their common predictor (H2,H3). In Model 3, the five SI dimensions are first-order latent variables that jointly predict OA (H4–H8). No single model contains both the path from SI to OA and the paths from SI to the two innovation dimensions; the direct paths to the dimensions therefore do not duplicate the path to the superordinate construct within one model, and no second-order factor with only two first-order factors had to be identified. The term “higher-order” is used conceptually: OA is the superordinate construct of the two innovation dimensions, but empirically it was not estimated as a second-order factor above item-level first-order factors.

**Fig. 1:**
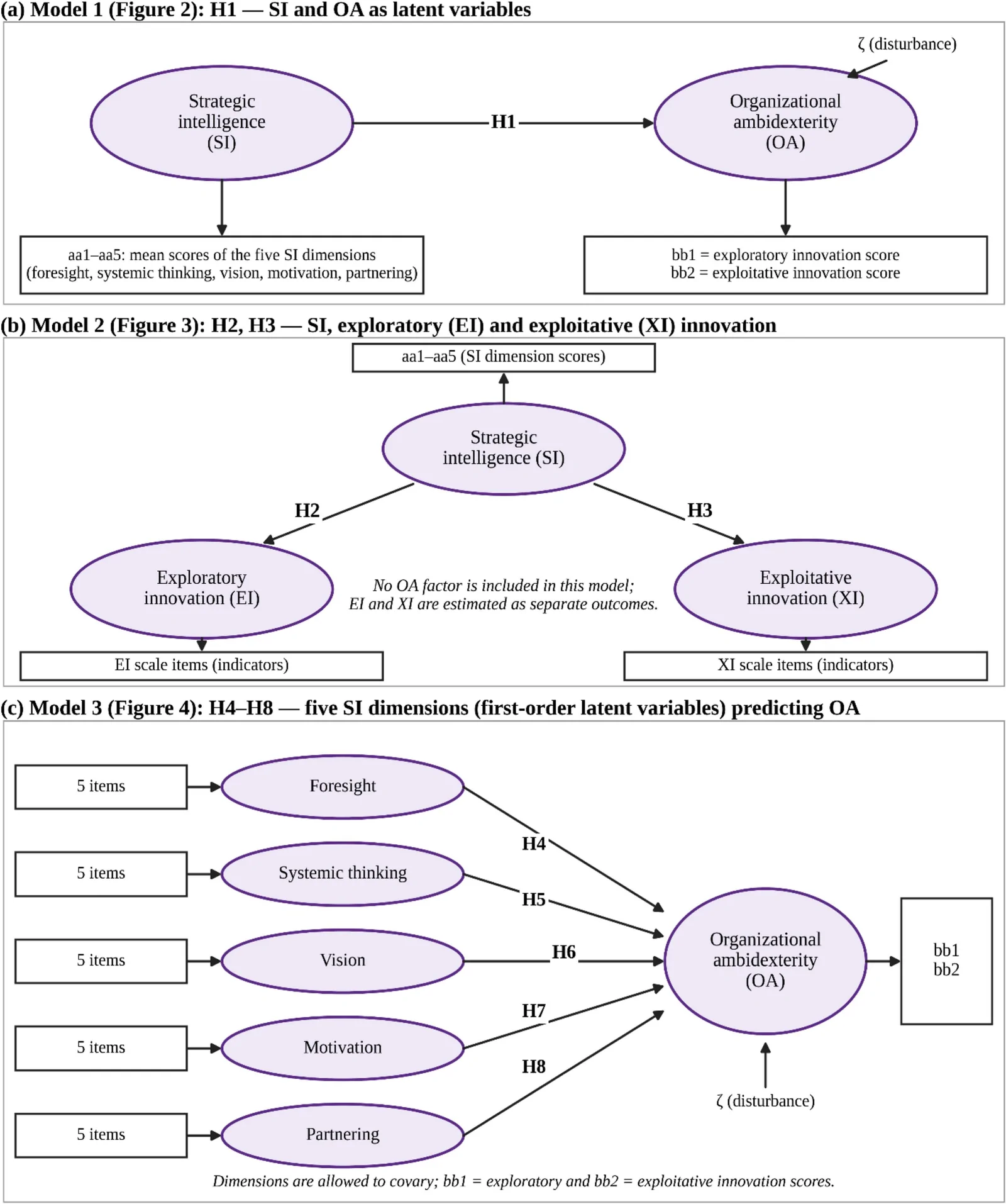
Conceptual model and measurement specification of the three structural models (Models 1–3) used to test H1–H8. Ellipses denote latent variables and rectangles observed indicators; ζ denotes the disturbance of the outcome. The models were estimated separately (see text).

This study aims to examine the association between strategic intelligence and organizational ambidexterity in a public healthcare network, treating both as organizational capabilities that may be relevant to health-system management. The study does not measure health-system performance or patient outcomes.

Fig. 1 illustrates the conceptual model and the measurement specification of the three structural models used to test the hypotheses.

This study focuses on the Health and Treatment Network of Ferdows County, Iran, as a theoretically justified empirical context for examining the SI-OA relationship. Although the data originate from a single public healthcare network, this setting shares features such as public financing, standardized national policies, and resource constraints with public healthcare organizations in other middle-income countries [42]; the degree of similarity was not empirically assessed. The network embodies archetypal post-pandemic challenges—digital transformation [43], workforce pressures, and resource scarcity—making it a “typical case” [44] of the VUCA conditions faced by healthcare systems [31]. Its manageable size (312 employees) enables rigorous quantitative analysis (SEM) with a complete sampling frame, facilitating “analytical generalization” (Yin, 2018). As a state-affiliated network, it offers institutional stability and reduced confounding, while its relevance to Iran's health policy context—characterized by reforms in health equity, resource allocation, and technological adoption [45]—provides locally valuable insights. Thus, while limited in scope, this single-case design is methodologically and theoretically justified, offering findings that may be informative for similar contexts, subject to replication.

The research hypotheses are presented as follows:

**Main Hypotheses:**

H1 Strategic intelligence (SI) is positively associated with organizational ambidexterity (OA) (as a higher-order composite construct) in the healthcare network of Ferdows County.

H2 Strategic intelligence (SI) is positively associated with exploratory innovation (one dimension of OA) in the healthcare network of Ferdows County.

H3 Strategic intelligence (SI) is positively associated with exploitative innovation (the second dimension of OA) in the healthcare network of Ferdows County.

**Sub-Hypotheses:**

Because OA is the superordinate construct of exploratory and exploitative innovation, it is also of interest which specific SI dimensions are most strongly associated with OA. The following sub-hypotheses test the association of each SI dimension with the overall OA construct:

H4 Foresight is positively associated with organizational ambidexterity in the healthcare network of Ferdows County.

H5 Systemic thinking is positively associated with organizational ambidexterity in the healthcare network of Ferdows County.

H6 Vision is positively associated with organizational ambidexterity in the healthcare network of Ferdows County.

H7 Motivation is positively associated with organizational ambidexterity in the healthcare network of Ferdows County.

H8 Partnering is positively associated with organizational ambidexterity in the healthcare network of Ferdows County.

It should be noted that the three main hypotheses (H1−H3) test the overall associations of SI with OA and its two dimensions. The five sub-hypotheses (H4–H8) decompose the SI construct to identify which specific capabilities (foresight, systemic thinking, vision, motivation, partnering) are most strongly associated with OA. This dual-level structure is used because (1) H1–H3 examine the overall association of SI with OA and its dimensions; and (2) H4–H8 provide more detailed information for theory development by identifying the SI dimensions with the strongest associations. The hypotheses are associational; the cross-sectional design does not permit causal inference.

Strategic intelligence and organizational ambidexterity are not health outcomes. They are organizational capabilities that were measured here through employees' perceptions in a public healthcare network. The present study did not measure healthcare delivery, quality, access, safety, continuity of care, preparedness, or population-health outcomes. Statements in this article about health-system performance or Sustainable Development Goal 3 therefore refer to potential implications to be examined in future research, not to results demonstrated by the present data.

## 2 Research methodology

This study is applied in terms of its purpose and descriptive of a correlational type in terms of its methodology. It is considered applied because the results are intended to improve organizational performance, and it is descriptive because it examines the current state of the association between strategic intelligence and organizational ambidexterity among the staff of the Health and Treatment Department in Ferdows County. Additionally, since the researcher aims to investigate the relationship between two variables, the research is categorized as correlational. Because the study focuses on organizational capabilities rather than clinical interventions, the unit of analysis is the healthcare employee's perception. The constructs are treated as potential organizational antecedents or enabling conditions of health-system functioning, not as demonstrated determinants of health-system performance; patient-level clinical outcomes were not measured.

$$n=\frac{N.{Z^{2}}_{\frac{\alpha }{2}}.p.q}{\ell^{2}\left(N-1\right)+{Z^{2}}_{\frac{\alpha }{2}}.p.q}$$

where *Z* is the z-score corresponding to the desired confidence level (1.96 for 95% confidence), *p* is the estimated proportion of the population with the characteristic of interest, *q* = 1 − *p* is the complementary proportion, and *d* is the acceptable margin of error. A value of *p* = 0.5 was used in this study as this represents the most conservative estimate of population variability—yielding the maximum possible sample size—and is standard practice when the true population proportion is unknown [46], [47]. With a 95% confidence level (*Z* = 1.96) and a 5% margin of error (*d* = 0.05), the initial sample size was calculated as *n* = 384.16. Since the statistical population of this study was 312 individuals (*N* = 312), the sample size was determined using the following population correction formula:

The Cochran formula with finite-population correction indicated a minimum of 172 respondents from the population of 312 employees. Because this calculation concerns the precision of a population proportion and does not address the requirements of structural equation modeling (SEM), the adequacy of *n* = 172 for the SEM analyses was evaluated separately and is interpreted cautiously. The evaluation is based on model complexity, expected effect size, sensitivity (minimum detectable effect) analysis, and a Monte Carlo simulation, not on the model-fit indices obtained after estimation. Model complexity. Three separate structural models were estimated (Fig. 2, Fig. 3, Fig. 4). The numbers of observed variables and estimated free parameters, counted from the model specifications, are given in Table 1. The ratio of cases to free parameters is approximately 11.5:1 for Model 1, which exceeds the 10:1 guideline, but only approximately 4.3:1 for Model 2 and 2.5:1 for Model 3, which fall below the commonly cited minimum of 5:1 [48]. Model 3, and to a lesser extent Model 2, are therefore based on a small sample relative to their complexity, and their estimates, especially for the individual SI dimensions, should be regarded as preliminary. Such ratios are heuristics; adequate sample size also depends on effect sizes, indicator reliability, and model complexity [49], [50]. Sensitivity analysis. A sensitivity analysis was conducted to determine the smallest effects that the achieved sample could detect (α = 0.05, two-sided, power = 0.80, *n* = 172), using exact noncentral distributions for the observed-variable regression equivalents of the structural paths. In a single-predictor model the smallest detectable standardized path is |β| ≈ 0.21 (R^2^ ≈ 0.045). In Model 3 the smallest detectable unique contribution of one SI dimension is f^2^ ≈ 0.047 (partial R^2^ ≈ 0.045), and the smallest detectable overall R^2^ of OA for the five predictors is ≈ 0.07 (f^2^ ≈ 0.077). Smaller effects could not be reliably detected, and latent-variable models with measurement error may require larger samples than these regression equivalents suggest. Monte Carlo simulation. To examine model stability at *n* = 172, a Monte Carlo simulation (2000 replications, maximum likelihood estimation, multivariate normal data) was run for Models 1 and 3, with population values set equal to the estimates shown in Fig. 2, Fig. 4 (in Model 3, inter-factor correlations were set to 0.31, the mean of the correlations in Table 5). Convergence was achieved in all replications, parameter bias was within ±5%, and the coverage of nominal 95% confidence intervals was 93.6–95.4%. Empirical power was practically 100% for the SI–OA path in Model 1 and for partnering in Model 3, but only about 61% for foresight, 53% for vision, and 16% and 13% for systemic thinking and motivation (Table 2). The non-significant results for the last two dimensions are therefore inconclusive rather than evidence of no association. Because population values were taken from the sample estimates, these figures are optimistic; Model 2 was not simulated. Software benchmark. The Soper [51] sample-size calculator for structural equation models (7 latent variables, 39 observed variables, anticipated effect f^2^ = 0.15, power = 0.80, α = 0.05) returned a minimum of 152 participants. This calculation was carried out after the sample size had been fixed and is reported only as a supplementary benchmark, not as an a priori determination; exceeding it does not establish adequacy for the more complex Models 2 and 3. No post-hoc (observed-effect) power analysis was used to justify the sample size.

**Fig. 2:**
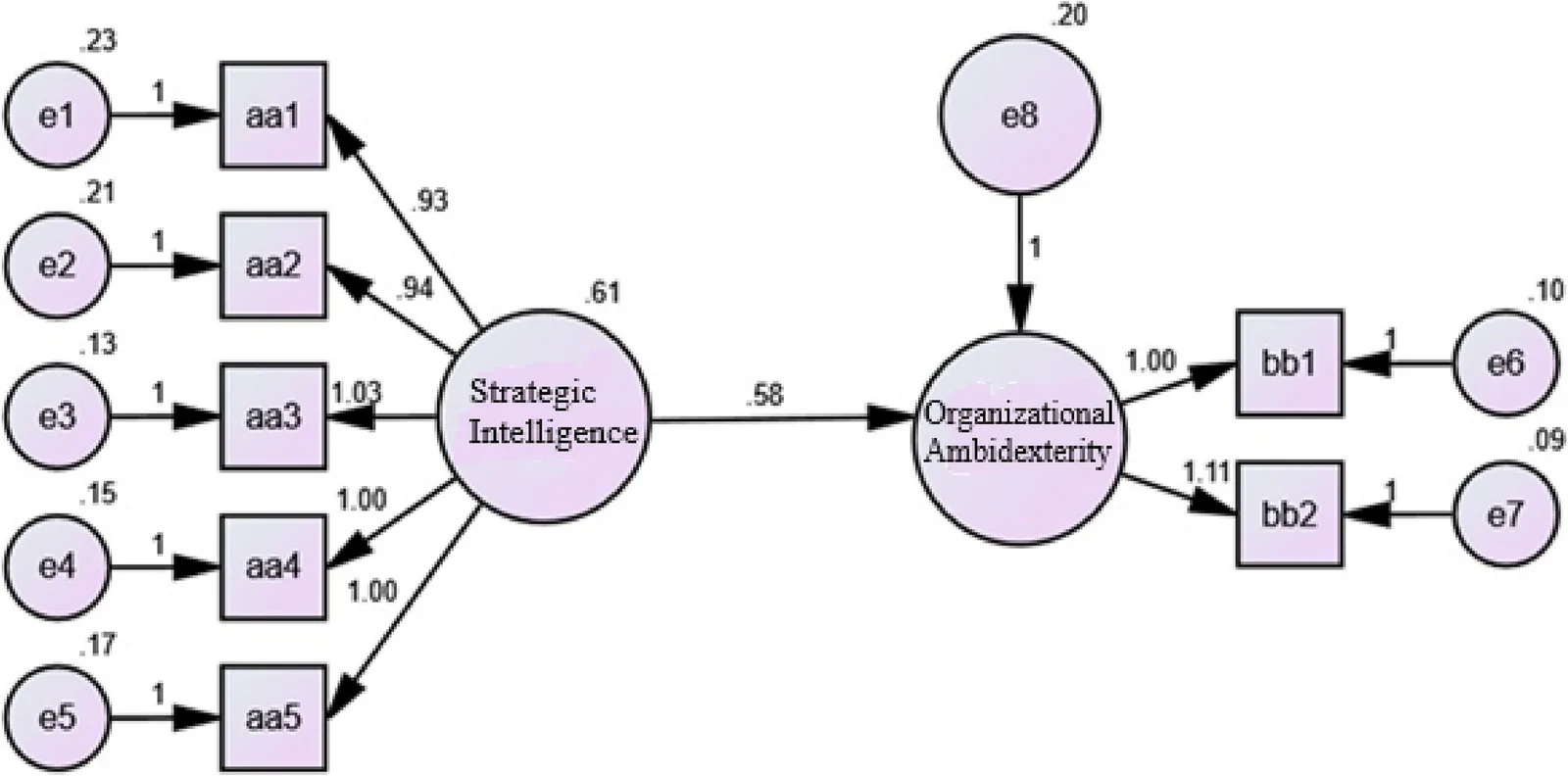
Structural model for testing the main hypothesis.

**Fig. 3:**
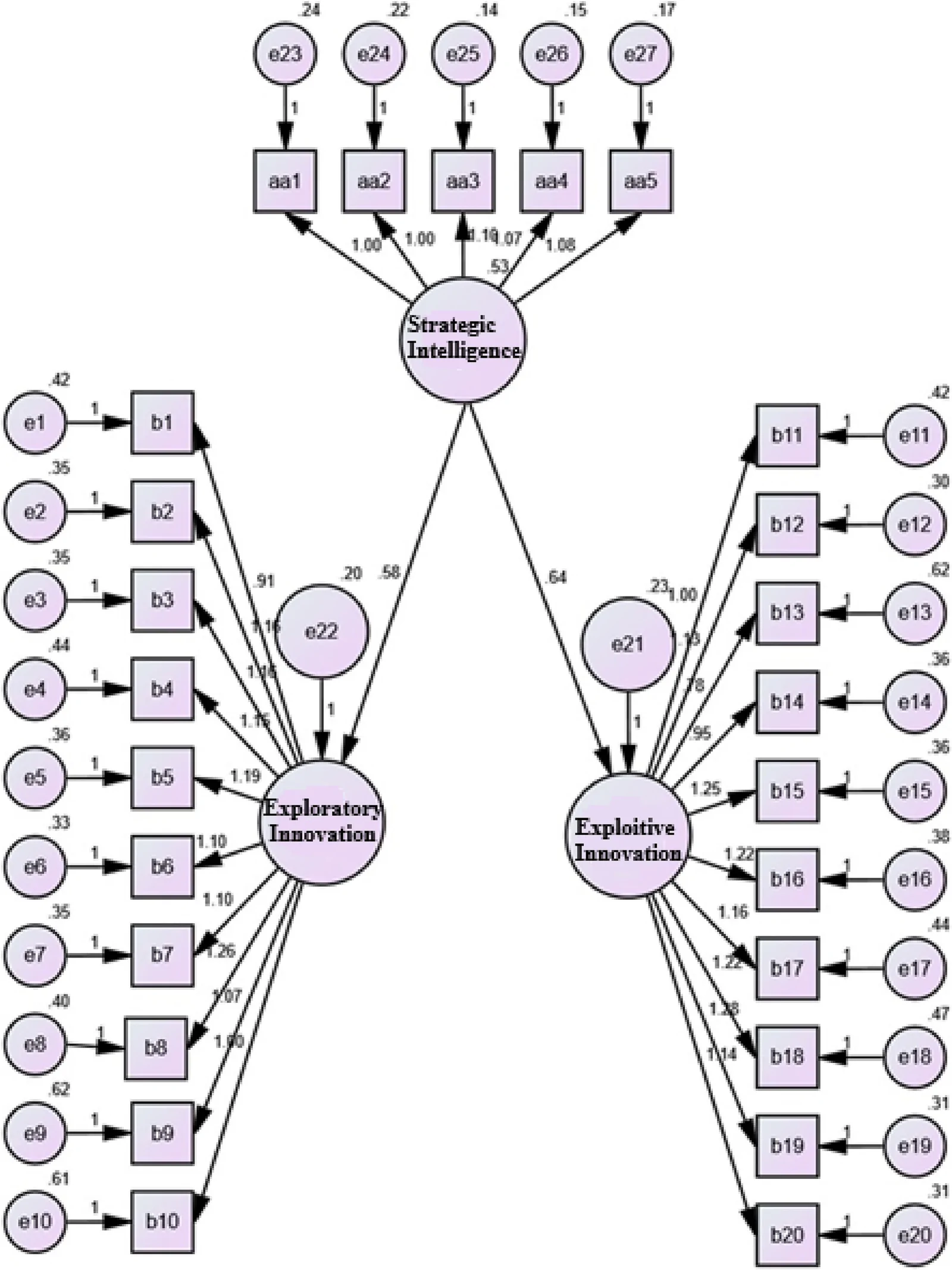
Structural model for testing the first and second sub-hypotheses.

**Fig. 4:**
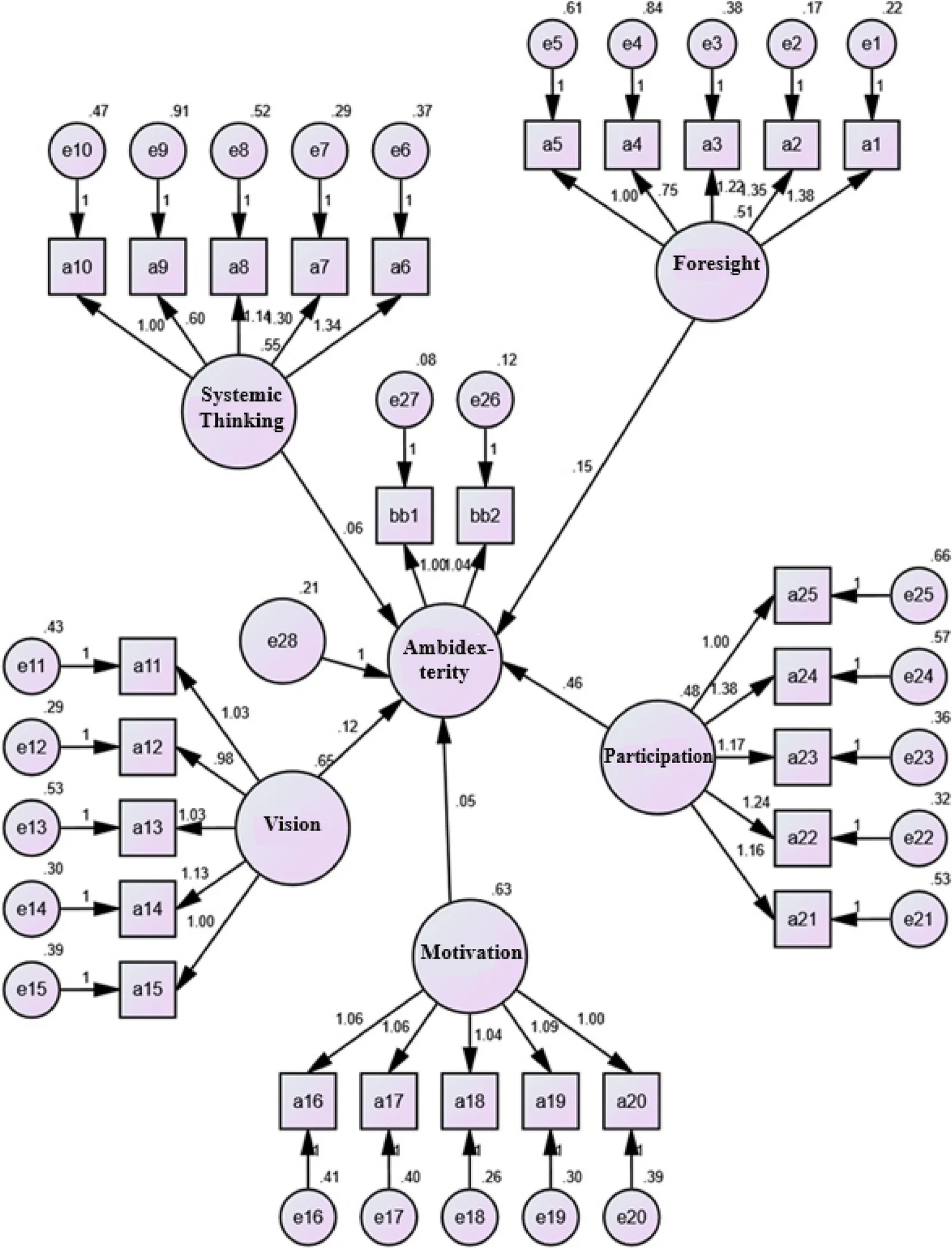
Structural model for testing sub-hypotheses 3 to 7.

**Table 1:** Model complexity and cases-to-parameter ratio for Models 1–3.

Model	Hypotheses	Observed indicators	Free parameters (approx.)	Cases-to-parameter ratio	Reported fit (CFI/TLI/RMSEA/CMIN·df^−1^)
1 (Fig. 2)	H1	7	15	11.5:1	0.982 / 0.971 / 0.076 / 2.45
2 (Fig. 3)	H2, H3	19	40	4.3:1	0.905 / 0.901 / 0.074 / 2.52
3 (Fig. 4)	H4–H8	27	69	2.5:1	0.911 / 0.903 / 0.068 / 2.67

Note. Free-parameter counts are derived analytically from each model's specification (loadings, error variances, factor variances/covariances, structural paths, and disturbance variances) and are approximate. The commonly cited minimum cases-to-parameter ratio is 5:1 [48].

**Table 2:** Monte Carlo simulation results at *n* = 172 (2000 replications, maximum likelihood).

Path (hypothesis)	Model	Population value (std. β)	Mean estimate	Bias (%)	95% CI coverage	Empirical power
SI → OA (H1)	1	0.580	0.581	0.1	95.3%	∼100%
Foresight → OA (H4)	3	0.167	0.167	1.4	94.4%	61.5%
Systemic thinking → OA (H5)	3	0.069	0.071	3.2	95.2%	16.0%
Vision → OA (H6)	3	0.150	0.150	0.1	93.6%	52.8%
Motivation → OA (H7)	3	0.062	0.059	−4.7	94.2%	13.0%
Partnering → OA (H8)	3	0.497	0.497	1.4	94.2%	∼100%

Note. Population values were fixed at the estimates shown in Fig. 2, Fig. 4 (Model 3 inter-factor correlations fixed at 0.31, the mean of the correlations in Table 5); all 2000 replications converged. Power below 80% indicates the achieved sample could not reliably detect an effect of that size, not that no association exists. Model 2 was not simulated.

**Study setting and transferability of findings:** The Health and Treatment Network of Ferdows County was selected because it represents a typical county-level public healthcare organization operating within the Iranian primary healthcare system. The network provides preventive, primary, and administrative healthcare services under standardized national policies established by the Ministry of Health and Medical Education. Because data were collected from employees of a single county-level healthcare network, the study should be regarded as a context-specific investigation of organizational behavior within one public healthcare organization. Although the structural relationships identified may be informative for comparable public healthcare settings, they should not be interpreted as directly generalizable to all healthcare organizations or healthcare systems.

The objective of this study was not to obtain a statistically representative sample of all healthcare organizations in Iran or other countries, but rather to examine the hypothesized relationships between strategic intelligence and organizational ambidexterity within a real-world public healthcare context. Consequently, the findings should be interpreted as evidence from a single organizational setting and should be transferred to other healthcare organizations only after careful consideration of differences in organizational structure, governance, resources, and healthcare delivery contexts. Sampling and response: Questionnaires were distributed to all 312 employees of the network; 172 usable responses were returned (response rate 55.1%). [AUTHORS: please confirm and insert the exact numbers invited, declined, and excluded, and the units/staff categories represented, if these differ from what is stated here.] Employees were not selected at random from the 312; all employees were invited, and the 172 respondents constitute those who chose to participate, so self-selection cannot be ruled out. Table 3 reports the occupational composition of respondents; administrative, health-unit, and treatment-unit staff were all represented, though not in equal numbers (Table 8).

**Table 3:** Demographic results.

Variables		Frequencies	Percentage
Gender	Male	64	37.21
	Female	108	62.79
Age	30 years or less	67	38.95
	31–40 years	76	44.19
	41–50 years	27	15.70
	51 years and above	2	1.16
Education	Associate's or less	19	11.05
	Bachelor's	110	63.95
	Master's and above	43	25.00
Working Experience	less than 5 year	66	38.37
	5–10 years	44	25.58
	11–15 years	24	13.95
	above 15 years	38	22.09
Position	Administrative	69	40.12
	Health	25	14.53
	Treatment	78	45.35

In terms of gender, the sample consisted of 64 male participants (37.21%) and 108 female participants (62.79%). In terms of age, 67 participants (38.95%) were under 30 years old, 76 participants (44.19%) were between 31 and 40 years old, 27 participants (15.70%) were between 41 and 50 years old, and 2 participants (1.16%) were over 50 years old. Regarding education, 19 participants (11.05%) had an associate degree or lower, 110 participants (63.95%) had a bachelor's degree, and 43 participants (25.00%) had postgraduate education. In terms of years of service, 66 participants (38.37%) had less than 5 years of experience, 44 participants (25.58%) had between 5 and 10 years of service, 24 participants (13.95%) had between 10 and 15 years of service, and 38 participants (22.09%) had more than 15 years of service. In terms of job placement, 69 participants (40.12%) worked in administrative units, 25 participants (14.53%) worked in health units, and 78 participants (45.35%) worked in medical units.

Strategic intelligence encompasses a systematic information-processing dimension and a cognitive capability dimension that enables anticipation of competitors' behavior and strategic adaptation [21]. Together, these dimensions support effective decision-making and strategic agility in dynamic environments. In this study, strategic intelligence is conceptualized through five key dimensions: foresight, which involves anticipating future trends and opportunities; systemic thinking, reflecting a holistic understanding of organizational and environmental interactions; vision, denoting a clear, long-term strategic direction; motivation, the drive to align organizational members toward strategic goals; and partnering, the ability to build strategic alliances and productive collaborative relationships with individuals, groups, and organizations [19]. Together, these components enable organizations to analyze competitive information effectively and adapt proactively in complex environments. Besides, organizational ambidexterity refers to an organization's ability to simultaneously pursue exploration (innovation, experimentation, and adaptation) and exploitation (refinement, efficiency, and execution of existing competencies). This dual capability enables firms to balance change and stability, thereby sustaining long-term performance in dynamic environments [52]. In the present study, organizational ambidexterity is conceptualized as the superordinate construct of two dimensions: exploratory innovation, which focuses on generating new knowledge, experimenting with novel ideas, and venturing into new domains; and exploitative innovation, which involves refining and utilizing existing capabilities to improve efficiency and performance. This conceptual structure was operationalized in three separate structural models rather than in a single second-order factor model (see Fig. 1 and the Model Specification subsection below). Balancing these dual innovation modes allows organizations to sustain adaptability and stability, critical for success in dynamic and uncertain contexts.

The data collection tools include standardized questionnaires on strategic intelligence by Maccoby [19] and organizational ambidexterity by Jansen et al. [53]. The strategic intelligence questionnaire consists of 25 questions based on a 5-point Likert scale. This questionnaire evaluates managers' strategic intelligence through its dimensions: questions 1 to 5 assess foresight, questions 6 to 10 assess systemic thinking, questions 11 to 15 assess vision formulation, questions 16 to 20 assess motivation, and questions 21 to 25 assess partnering [19]. The second questionnaire evaluates organizational ambidexterity and includes 14 questions based on a Likert scale, divided into two dimensions: exploratory innovation (questions 1 to 7) and exploitative innovation (questions 8 to 14) [53]. The Strategic Intelligence Questionnaire was originally developed and validated with managers [19]; in this study it was administered to all healthcare employees, including non-managerial staff, because the items ask about the respondent's own perceptions and behaviors (e.g., anticipating change, understanding how one's work fits the larger system) rather than about managerial duties specifically, and comparable adaptations to non-managerial healthcare staff have been used in prior organizational research. This extension of the instrument's use beyond its original managerial validation sample is nonetheless a measurement limitation that is revisited in the Limitations subsection.

Because the original measurement instruments developed by Maccoby [19] and Jansen et al. [53] were published in English, they were adapted into Persian following a structured forward-translation and back-translation procedure. Two bilingual translators with graduate training in health services management independently produced forward translations from English to Persian. A reconciled Persian version was produced by consensus discussion between the two translators and a third bilingual reviewer. This reconciled version was then back-translated into English by a fourth bilingual translator who had not seen the original instrument, and the back-translation was compared with the original English items to identify discrepancies in meaning. Three academic experts in healthcare management and organizational behavior then reviewed the reconciled Persian version for clarity, relevance, and cultural appropriateness; their feedback led to minor wording changes to two items. The revised Persian version was piloted with 30 employees not included in the main sample (see also the item pre-testing described under Common Method Bias Assessment), and no further changes were required after piloting. A formal quantitative content-validity index was not computed; content adequacy was judged qualitatively by the expert panel. This is noted as a limitation.

Content validity was judged adequate based on the expert panel review described above, together with the questionnaires' standardized and previously validated nature. Cronbach's alpha was used to measure the reliability of the questionnaires. The reliability of the strategic intelligence questionnaire was calculated as 0.885, and the reliability of the organizational ambidexterity questionnaire was calculated as 0.925. Additionally, the reliability of each dimension of the questionnaires was also calculated, all exceeding 0.7, thereby confirming their reliability.

For data analysis, descriptive and inferential statistics (*t*-tests and structural equation modeling) were utilized, using SPSS and AMOS software. AMOS was selected for this study due to its robust capabilities in structural equation modeling (SEM), which aligns with our research objectives. SEM allows for the simultaneous analysis of multiple relationships between latent variables (e.g., strategic intelligence dimensions and organizational ambidexterity) while accounting for measurement error. This approach is particularly suitable for testing complex theoretical models with interdependent hypotheses, as in our study. AMOS also provides comprehensive goodness-of-fit indices, enabling rigorous validation of both the measurement and structural models.

In the following section, the data analysis techniques used in this research are explained.

### 2.1 Data analysis techniques

The primary analytical approach for hypothesis testing was Maximum Likelihood (ML) estimation, as the model contains only direct paths and no mediation effects. Bootstrapping, where used, served only as a supplemental robustness check. To ensure the robustness of the findings, multiple data analysis techniques were employed with particular attention to potential threats to validity, including common method bias, which was assessed using both statistical and procedural remedies. The data analysis was conducted in three stages using SPSS (version 26) and AMOS (version 24) software:

1. Descriptive statistics

• Frequency distributions and percentages were calculated for demographic variables (e.g., age, gender, education).
• Mean and standard deviation were computed for all Likert-scale items.

2. Confirmatory Factor Analysis (CFA):

• Used to validate the measurement model for both constructs (strategic intelligence and organizational ambidexterity).
• Assessed model fit using indices: CFI (>0.90), TLI (>0.90), RMSEA (<0.08), and CMIN/DF (<3).
• **Convergent validity assessment:** Convergent validity was assessed using three criteria [54], [55]: (a) standardized factor loadings >0.50 and statistically significant (*p* < 0.001); (b) Average Variance Extracted (AVE) > 0.50 for each construct, indicating that the construct explains more than 50% of the variance in its indicators; and (c) Composite Reliability (CR) > 0.70, indicating internal consistency reliability. Cronbach's alpha (> 0.70) was also calculated as a supplementary measure.
• **Discriminant validity assessment:** Discriminant validity was assessed using two criteria: (a) the Fornell-Larcker criterion, where the square root of AVE for each construct must exceed its correlations with all other constructs; and (b) the Heterotrait-Monotrait (HTMT) ratio of correlations, with values below 0.85 indicating adequate discriminant validity [56].

3. Structural Equation Modeling (SEM)

• Tested hypotheses by analyzing path coefficients (β) and their significance (*p* < 0.05).
• Evaluated direct effects of strategic intelligence on organizational ambidexterity, exploratory innovation, and exploitative innovation.
• Reported goodness-of-fit indices (CFI, TLI, RMSEA, CMIN/DF) for the structural model.

**Common Method Bias Assessment:** Given that all data were collected from a single source (self-report questionnaires) at a single time point, the potential for common method bias (CMB) was a concern. Common method bias refers to systematic variance shared among variables due to the measurement method rather than the constructs they represent [57]. To address this, two complementary approaches were employed:

• **Harman's Single-Factor Test:**

Harman's single-factor test was conducted as an initial diagnostic assessment of common method bias [58], [59]. All 39 observed indicator variables (25 strategic intelligence items and 14 organizational ambidexterity items) were entered into an exploratory factor analysis (EFA) using principal component analysis with unrotated solution. The analysis extracted seven factors with eigenvalues exceeding 1.0, together accounting for 68.4% of the total variance. The first unrotated factor accounted for only **24.7%** of the total variance, which is substantially below the conservative threshold of 50% [57]. This result suggests that common method bias is not a pervasive issue in the dataset, as no single factor emerged to account for the majority of covariance among the measures.

• **Marker Variable Approach (Partial Correlation Procedure):**

To provide a more rigorous assessment, a marker variable approach was employed following established guidelines [60], [61]. A theoretically unrelated marker variable—“attitude toward computer technology”—was included in the survey. This three-item measure (α = 0.76) assessed respondents' general attitudes toward workplace technology, which was theoretically uncorrelated with strategic intelligence and organizational ambidexterity. The marker items were included in the questionnaire at the design stage, prior to data collection, and were administered alongside the SI and OA items in the same survey. Attitude toward workplace technology was selected as the marker because it concerns evaluative beliefs about digital tools rather than the cognitive and behavioral strategic capabilities captured by the SI and OA scales; however, we acknowledge that attitudes toward technology are plausibly related to organizational innovation more broadly, so this marker provides only a partial, not a definitive, check on common method bias, and the results below should be read with that caveat. This choice of marker follows precedent in prior CMB studies that treat technology attitudes as conceptually distinct from the organizational-behavior constructs of substantive interest [57], although, as noted above, this distinctiveness is an assumption rather than an empirically demonstrated property of this specific marker in this sample.

The smallest positive correlation between the marker variable and any of the study's key constructs was *r* = 0.04 (between the marker and foresight), which was not statistically significant (*p* = 0.52). Following the partial correlation adjustment procedure recommended by Lindell and Whitney [60], the adjusted correlations were calculated by partialling out the marker variable from the relationships among the focal constructs. All originally significant correlations among the study variables remained significant (*p* < 0.05) after this adjustment, with negligible changes in the magnitude of correlation coefficients (average reduction = 0.03). This further confirms that common method bias does not substantially threaten the validity of the findings.

**Procedural Remedies:** Beyond statistical assessments, several procedural measures were implemented during the survey administration to minimize common method bias, consistent with recommendations by Podsakoff et al. [57]:

• **Anonymity and confidentiality:** Respondents were assured of complete anonymity and informed that there were no right or wrong answers, reducing social desirability bias.
• **Psychological separation:** Data were collected at a single time point; no temporal separation of measurement was implemented. To reduce consistency artifacts, the questionnaire was organized into distinct sections (strategic intelligence items in Section A; organizational ambidexterity items in Section B), which is a form of psychological, not temporal, separation between the constructs.
• **Clear instructions:** Respondents were informed that the survey was part of an academic study with no organizational consequences, reducing evaluation apprehension.
• **Item clarity:** The questionnaire items were carefully worded to avoid ambiguity and double-barreled phrasing, and were pre-tested with 30 employees to ensure comprehension.

Collectively, the statistical tests (Harman's single-factor test and marker variable analysis) and procedural remedies are reassuring but cannot rule out common method bias, which remains a limitation of any single-source, single-time-point, self-report design; the associations reported below should be interpreted with this caveat in mind.

**Statistical Power Assessment:** To directly address the adequacy of the sample size for detecting the hypothesized effects, a post-hoc statistical power analysis was conducted using the Soper [51] SEM sample size calculator. With df = 89, an anticipated medium effect size (f^2^ = 0.15), a significance level of α = 0.05, and the achieved sample of *n* = 172, the estimated statistical power exceeded 0.85 for the main structural paths. This surpasses the conventional 0.80 threshold [62], confirming that the study is sufficiently powered to validate the hypothesized relationships.

Although a bootstrapping procedure (2000 samples) was conducted during the analytical phase, this procedure was used solely as a supplemental robustness check to assess the stability of the standard errors and confidence intervals for the direct path coefficients, and not to test indirect or mediation effects. Since the study's conceptual model does not hypothesize any mediation relationships, bootstrapping was not a primary analytical requirement. The Maximum Likelihood estimation results, reported in the Findings section, are the primary basis for hypothesis testing. The bootstrapping results confirmed the stability of the ML estimates, with bias-corrected confidence intervals for all significant paths not crossing zero, providing additional assurance regarding the reliability of the parameter estimates.

**Additional tests:**

• Kolmogorov-Smirnov test confirmed normality of data distribution (*p* > 0.05).
• One-way ANOVA compared variable means across demographic groups (e.g., gender, work experience).

## 3 Findings and discussions

According to the frequency distribution of participants in Table 3, the sample under study consists of 37.21% males and 62.79% females. The highest percentage in the age variable of respondents is in the age group of 31 to 40 years, accounting for 44.19%, while in terms of education, the highest percentage belongs to the Bachelor's degree level, with 63.95%. Additionally, 45.35% of the sample are employed in the healthcare unit.

Before testing the research hypotheses, the distributional assumptions required for Maximum Likelihood (ML) estimation in SEM were examined. The Kolmogorov-Smirnov (K—S) test was used as an initial screen: the null hypothesis of a normal distribution was retained for strategic intelligence (test statistic = 0.930, *p* = 0.328) and for organizational ambidexterity (test statistic = 0.851, *p* = 0.464), and all constituent dimensions likewise had K—S significance levels above 0.05. A non-significant K—S test indicates that univariate normality could not be rejected; it does not by itself establish that the multivariate-normality assumption of ML estimation is satisfied, and item-level skewness, kurtosis, and a multivariate normality statistic such as Mardia's coefficient were not computed for this analysis. This is noted as a limitation of the reported assumption checks; because ML estimates and standard errors can be sensitive to multivariate non-normality, the parameter estimates reported below should be interpreted with this caveat, and future reporting of this study should include Mardia's coefficient and, if multivariate normality is violated, a robust (e.g., Satorra-Bentler-corrected) or bootstrap re-estimation.

This study employed structural equation modeling (SEM) using IBM SPSS AMOS (version 24) to test the research hypotheses. The measurement model was first evaluated through confirmatory factor analysis (CFA) to ensure that the observed variables accurately reflected the constructs of strategic intelligence and organizational ambidexterity. Prior to testing the structural hypotheses, the measurement model was rigorously evaluated for convergent and discriminant validity, as these are essential prerequisites for SEM-based studies [55]. Convergent validity was assessed using three established criteria: standardized factor loadings, Average Variance Extracted (AVE), and Composite Reliability (CR).

For the Strategic Intelligence Questionnaire [19], all standardized factor loadings exceeded 0.50 and were statistically significant (*p* < 0.001), ranging from 0.62 to 0.89 across the five dimensions (foresight, systemic thinking, vision, motivation, partnering). The AVE values for each dimension were: foresight = 0.56, systemic thinking = 0.51, vision = 0.62, motivation = 0.58, and partnering = 0.68, all exceeding the recommended threshold of 0.50 [54]. Composite Reliability (CR) values were: foresight = 0.84, systemic thinking = 0.82, vision = 0.88, motivation = 0.86, and partnering = 0.91, all exceeding the 0.70 threshold [55]. These results confirm adequate convergent validity for the strategic intelligence construct.

For the Organizational Ambidexterity Questionnaire [53], all standardized factor loadings exceeded 0.50 and were statistically significant (*p* < 0.001), ranging from 0.58 to 0.91 across the two dimensions (exploratory innovation and exploitative innovation). The AVE values were: exploratory innovation = 0.59 and exploitative innovation = 0.63, both exceeding the 0.50 threshold. Composite Reliability (CR) values were: exploratory innovation = 0.89 and exploitative innovation = 0.92, both exceeding the 0.70 threshold. These results confirm adequate convergent validity for the organizational ambidexterity construct.

A summary of the convergent validity metrics is presented in Table 4.

**Table 4:** Convergent validity results (AVE and CR values).

Construct	Dimension	No. of Items	AVE	CR	Cronbach's α
Strategic Intelligence	Foresight	5	0.56	0.84	0.83
	Systemic Thinking	5	0.51	0.82	0.81
	Vision	5	0.62	0.88	0.87
	Motivation	5	0.58	0.86	0.85
	Partnering	5	0.68	0.91	0.90
Organizational Ambidexterity	Exploratory Innovation	7	0.59	0.89	0.88
	Exploitative Innovation	7	0.63	0.92	0.91

Discriminant validity was assessed using two methods: the Fornell-Larcker criterion and the Heterotrait-Monotrait (HTMT) ratio of correlations.

**Fornell-Larcker Criterion:** As shown in Table 5, the square root of AVE for each construct (diagonal values) exceeds the inter-construct correlations (off-diagonal values), confirming discriminant validity.

**Table 5:** Fornell-Larcker criterion results.

Construct	1	2	3	4	5	6	7
1. Foresight	**0.748**						
2. Systemic Thinking	0.313	**0.714**					
3. Vision	0.287	0.295	**0.787**				
4. Motivation	0.302	0.278	0.310	**0.762**			
5. Partnering	0.345	0.321	0.339	0.354	**0.825**		
6. Exploratory Innovation	0.421	0.398	0.412	0.385	0.502	**0.768**	
7. Exploitative Innovation	0.435	0.402	0.428	0.391	0.518	0.512	**0.794**

As shown in Table 6, all HTMT ratios were below the conservative threshold of 0.85 [56], ranging from 0.61 to 0.78, further confirming discriminant validity.

**Table 6:** Heterotrait-Monotrait (HTMT) ratios.

Construct Pair	HTMT Ratio
Foresight ↔ Systemic Thinking	0.65
Foresight ↔ Vision	0.63
Foresight ↔ Motivation	0.67
Foresight ↔ Partnering	0.71
Systemic Thinking ↔ Vision	0.64
Systemic Thinking ↔ Motivation	0.62
Systemic Thinking ↔ Partnering	0.68
Vision ↔ Motivation	0.69
Vision ↔ Partnering	0.72
Motivation ↔ Partnering	0.74
Exploratory Innovation ↔ Exploitative Innovation	0.78
Strategic Intelligence (all dimensions) ↔ Exploratory Innovation	0.65–0.73
Strategic Intelligence (all dimensions) ↔ Exploitative Innovation	0.68–0.76

In summary, both convergent validity (AVE > 0.50; CR > 0.70) and discriminant validity (Fornell-Larcker criterion met; HTMT <0.85) were established for all constructs in the measurement model, confirming that the translated instruments are psychometrically robust in the Iranian public-sector healthcare context.

Model fit indices confirmed the adequacy of the measurement model. The measurement-model factor loadings were statistically significant, and the model fit indices were within acceptable ranges, supporting the suitability of the structural model for hypothesis testing, and fit indices were within acceptable ranges, supporting the suitability of the structural model for hypothesis testing.

Hypothesis testing was conducted through the following steps:

**Measurement model validation:** Confirmatory Factor Analysis (CFA) was performed to ensure the observed variables adequately represented the latent constructs. All factor loadings exceeded 0.5, and model fit indices met thresholds (CFI > 0.90, TLI > 0.90, RMSEA <0.08, CMIN/DF < 3).

**Structural model testing:** Prior to structural path testing, the measurement model was evaluated for convergent and discriminant validity using: (a) standardized factor loadings >0.50; (b) Average Variance Extracted (AVE) > 0.50; (c) Composite Reliability (CR) > 0.70; (d) the Fornell-Larcker criterion (square root of AVE > inter-construct correlations); and (e) HTMT ratios <0.85 [56]. Path coefficients (β) and their significance levels (*p* < 0.05) were examined to test the hypotheses. Given that the proposed model contains only direct effects (no indirect or mediation paths are hypothesized), standard Maximum Likelihood (ML) estimation was used for parameter estimation in AMOS. ML estimation is appropriate for normally distributed data and provides unbiased parameter estimates with consistent standard errors [49]. Bootstrapping (2000 samples) was used only as a supplementary robustness check to confirm the stability of standard errors and confidence intervals. All hypothesized relationships are direct paths from strategic intelligence (and its dimensions) to organizational ambidexterity (and its dimensions). No mediation hypotheses were tested in this study.

**Effect size interpretation:** Standardized β values were interpreted as small (0.10), medium (0.30), or large (0.50) effects, per Cohen [62]. For instance, the effect of strategic intelligence on organizational ambidexterity (β = 0.580) was large and statistically significant (*p* < 0.001), supporting Hypothesis 1.

Fig. 2 shows the structural equation model of the research based on the main hypothesis. Next, the goodness-of-fit of the model must be examined. The Comparative Fit Index (CFI) and Tucker-Lewis Index (TLI) values were obtained as 0.982 and 0.971, respectively. The ideal value for these indices is greater than 0.90. Additionally, the Root Mean Square Error of Approximation (RMSEA) and the Chi-Square to Degrees of Freedom ratio (CMIN/DF) were calculated as 0.076 and 2.449, respectively. The desired values for these indices are less than 0.08 and less than 3. Therefore, all indices fall within an acceptable range, indicating that the model fits well.

To further contextualize the adequacy of the sample relative to model complexity, the structural model contained 89 degrees of freedom (df) and 65 estimated free parameters based on 39 observed indicator variables. The sample-to-parameter ratio was approximately 2.6:1 (172:65), while the sample-to-degrees-of-freedom ratio was approximately 1.93:1 (172:89) [48]. Although the sample-to-parameter ratio does not meet the commonly cited 5:1 rule of thumb, the sample size was considered adequate for the present SEM analysis based on the model's acceptable fit indices and the additional power analysis reported above. These results provide overall support for the adequacy of the sample for the structural equation modeling.

Model 1 (Fig. 2) tested H1, the association of strategic intelligence with the OA latent variable. The standardized structural path coefficient was β = 0.580 (unstandardized B = 0.580, SE = 0.061, z = 9.526, *p* < 0.001, 95% CI [0.461, 0.699]). Because OA has a single predictor in this model, the squared multiple correlation for OA equals the squared standardized path coefficient: R^2^ = 0.336. That is, strategic intelligence accounted for approximately 33.6% of the variance in organizational ambidexterity in this model, not 58.0% as the standardized coefficient alone might suggest; β and R^2^ are numerically different quantities and are reported separately here (Table 7 lists SE, 95% CI, and R^2^ for all reported paths). Model fit indices were within conventional ranges: CFI = 0.982, TLI = 0.971, RMSEA = 0.076, CMIN/DF = 2.449.

**Table 7:** Standardized structural path estimates, standard errors, 95% confidence intervals, and R^2^ across Models 1–3.

Path	Model	Standardized β	SE	95% CI	R^2^ of outcome	p
SI → OA (H1)	1	0.580	0.061	[0.461, 0.699]	0.336	< 0.001
SI → Exploratory innovation (H2)	2	0.585	≈ 0.061	n/r	0.342	< 0.001
SI → Exploitative innovation (H3)	2	0.639	≈ 0.061	n/r	0.408	< 0.001
Foresight → OA (H4)	3	0.152	n/r	n/r	–	< 0.05
Systemic thinking → OA (H5)	3	0.067	n/r	n/r	–	0.300 (ns)
Vision → OA (H6)	3	0.120	n/r	n/r	–	< 0.05
Motivation → OA (H7)	3	0.058	n/r	n/r	–	0.371 (ns)
Partnering → OA (H8)	3	0.458	n/r	n/r	≈ 0.45–0.50⁎	< 0.001

⁎ Because the five Model 3 predictors are correlated (Table 5), R^2^ for OA cannot be recovered from the individual path coefficients alone; the range shown is implied by the reported paths and the observed inter-factor correlations and should be confirmed against the exact AMOS squared-multiple-correlation output. SE and 95% CI for individual Model 2 and Model 3 paths (“n/r”) were not reported in the original AMOS output available for this revision and should be added from the AMOS text output before final publication. ns = not significant.

Model 2 (Fig. 3) tested H2 and H3, the associations of strategic intelligence with exploratory and exploitative innovation as separate latent variables. Model fit indices were CFI = 0.905, TLI = 0.901, RMSEA = 0.074, CMIN/DF = 2.52. Strategic intelligence was positively associated with exploratory innovation (β = 0.585, SE ≈ 0.061, *p* < 0.001; R^2^ = 0.342, i.e., approximately 34.2% of variance explained) and with exploitative innovation (β = 0.639, SE ≈ 0.061, *p* < 0.001; R^2^ = 0.408, i.e., approximately 40.8% of variance explained). As in Model 1, R^2^ is reported as the square of the standardized path coefficient because each outcome has a single predictor in this model; it is not equal to β.

Model 3 (Fig. 4) tested H4–H8, the associations of the five SI dimensions, modeled as correlated first-order latent variables, with OA. Model fit indices were CFI = 0.911, TLI = 0.903, RMSEA = 0.068, CMIN/DF = 2.67. Foresight (H4: β = 0.152, *p* < 0.05), vision (H6: β = 0.120, *p* < 0.05), and partnering (H8: β = 0.458, *p* < 0.001) were positively associated with OA; systemic thinking (H5: β = 0.067, z = 1.036, *p* > 0.05) and motivation (H7: β = 0.058, z = 0.894, *p* > 0.05) were not statistically significant. H5 and H7 are therefore not supported by these data, while H4, H6, and H8 are supported. Because the five predictors are correlated (Table 5), R^2^ for OA in this model is not the sum of the squared path coefficients; based on the reported path coefficients and the observed inter-factor correlations, the implied R^2^ is approximately 0.45–0.50, but the exact AMOS squared-multiple-correlation value for OA in this model should be verified against the AMOS output and reported exactly. Table 7 summarizes β, SE, 95% CI, and R^2^ for the paths in all three models; readers requiring the complete AMOS text output are referred to the corresponding author.

As a secondary, exploratory analysis not linked to the prespecified hypotheses, a series of one-way ANOVA tests examined whether perceptions of SI and OA varied across demographic and job-unit groups. The results, presented in Table 8, reveal a consistent pattern across all variables.

**Table 8:** One-Way ANOVA results comparing variable means across demographic groups.

Demographic Variable	Group	n	Mean (SI)	Mean (OA)	F-Statistic (SI)	F-Statistic (OA)	*p*-value (SI)	p-value (OA)	Significant?
Gender	Male	64	3.82	3.75	0.842	0.691	0.361	0.408	No
	Female	108	3.74	3.68					
Age	≤30 years	67	3.78	3.72	0.523	0.612	0.672	0.545	No
	31–40 years	76	3.81	3.70					
	41–50 years	27	3.69	3.65					
	≥51 years	2	3.85	3.80					
Education	Associate or less	19	3.71	3.62	1.024	0.893	0.382	0.415	No
	Bachelor's	110	3.79	3.71					
	Master's or above	43	3.76	3.68					
Work Experience	<5 years	66	3.80	3.73	0.712	0.654	0.548	0.581	No
	5–10 years	44	3.76	3.70					
	11–15 years	24	3.72	3.64					
	>15 years	38	3.83	3.76					
Job Unit	Administrative	69	3.92	3.88	**6.734**	**7.215**	0.001*	0.001*	**Yes**
	Health	25	3.58	3.45					
	Treatment	78	3.71	3.62					

As shown in Table 8, no significant differences were found across gender, age, education, or work experience groups for either SI or OA perceptions. A statistically significant difference was observed across job units for both SI (F(2,169) = 6.734, *p* = 0.001, η^2^ ≈ 0.07) and OA (F(2,169) = 7.215, *p* = 0.001, η^2^ ≈ 0.08). Because the three job-unit groups are unequal in size (administrative *n* = 69, health unit *n* = 25, treatment unit *n* = 78), Levene's test for homogeneity of variance should be examined before interpreting Tukey HSD post-hoc comparisons; this statistic was not computed for the present analysis and its absence is noted as a limitation of this exploratory comparison. If Levene's test indicates heterogeneity of variance in the final dataset, a variance-robust post-hoc procedure (e.g., Games-Howell) should be substituted for Tukey HSD. Subject to this caveat, the Tukey HSD comparisons indicated the following pattern:

**Administrative staff** reported the highest perceptions of both SI (M = 3.92) and OA (M = 3.88).

**Health unit staff** reported the lowest perceptions of both SI (M = 3.58) and OA (M = 3.45).

**Treatment unit staff** fell in the middle for both SI (M = 3.71) and OA (M = 3.62).

The differences between administrative staff and health unit staff were significant for both variables (*p* < 0.01), while differences between administrative and treatment units approached significance (*p* = 0.06 for SI; *p* = 0.07 for OA; neither difference reached the conventional 0.05 threshold).

The significant differences in perceptions of strategic intelligence (SI) and organizational ambidexterity (OA) across job units may be explained by differences in role proximity to strategic decision-making, access to strategic information, organizational culture, psychological ownership, and job demands. Administrative staff are generally more involved in strategic planning, resource allocation, policy development, and decision-making, giving them greater exposure to foresight, vision formulation, and partnering processes and consequently higher awareness of SI and OA capabilities [21], [23]. In contrast, health and treatment staff primarily focus on frontline service delivery and patient care and may have less access to strategic discussions, while functional silos and distinct professional priorities can further limit strategic information sharing and interdisciplinary collaboration [22], [30]. Moreover, greater involvement in organizational decisions may strengthen administrative staff's psychological ownership, whereas frontline staff face competing demands such as patient care, emergency response, and regulatory requirements, limiting their engagement with strategic initiatives. These explanations are plausible interpretations offered for this secondary, exploratory comparison; they were not tested directly in this study and should be treated as hypotheses for future research rather than as findings. As with the main structural results, any implications for healthcare delivery are indirect and were not measured here.

### 3.1 Theoretical implications

The organizational capabilities examined here, strategic intelligence and organizational ambidexterity, have been discussed in the broader literature as potential enabling conditions for evidence-based practice, multidisciplinary coordination, resource allocation, and continuity of services (see Section 1). The present study provides evidence on the association between SI and OA as organizational-level perceptions; it does not test whether these organizational capabilities translate into the downstream healthcare-delivery outcomes that the literature associates with them, and no such causal or mediating pathway is claimed here.

This study set out to examine the association between strategic intelligence (SI) and organizational ambidexterity (OA) in a healthcare setting, a sector that must balance exploiting existing capabilities with exploring innovations [2]. Modeling OA as the superordinate construct of exploratory and exploitative innovation, our results are consistent with all three main hypotheses: SI was positively associated with the overall OA construct (H1: β = 0.580, R^2^ = 0.336, *p* < 0.001), with exploratory innovation (H2: β = 0.585, R^2^ = 0.342, *p* < 0.001), and with exploitative innovation (H3: β = 0.639, R^2^ = 0.408, *p* < 0.001). These three associations were estimated in three separate structural models (Fig. 2, Fig. 3, Fig. 4; see Model Specification), not within a single hierarchical model, so they should be read as complementary rather than as nested components of one estimated structure. Among the SI dimensions (Model 3), partnering (β = 0.458) and foresight (β = 0.152) showed the strongest positive associations with OA, consistent with the broader idea that these capabilities may help healthcare organizations navigate the exploration-exploitation trade-off [7]; this study establishes association, not the mechanism by which that trade-off is navigated.

Theoretically, this study strengthens the connection between SI and OA, particularly in high-stakes, service-oriented industries. The present research contributes to the literature on strategic intelligence (SI), organizational ambidexterity (OA), and innovation management in healthcare organizations in several ways:

**Advancing the Strategic Intelligence–Organizational Ambidexterity Nexus:** Prior research has largely treated strategic intelligence and organizational ambidexterity as independent constructs without fully exploring their interdependencies in service industries like healthcare. This study addresses that gap with cross-sectional evidence that SI is positively associated with both exploratory and exploitative innovation.

**Extending Ambidexterity Research into Healthcare:** While organizational ambidexterity has been widely studied in the private sector, its implications for healthcare organizations remain underexplored. This research provides evidence consistent with the view that healthcare institutions benefit from balancing exploration (e.g., adopting new medical technologies) and exploitation (e.g., refining existing procedures) to enhance adaptability and efficiency.

**Unpacking the Role of SI Dimensions in OA:** Unlike previous research that treats SI as a single construct, this study decomposes it into foresight, systemic thinking, vision, motivation, and partnering. The findings suggest that partnering and foresight are the dimensions most strongly associated with OA in this sample. This nuanced understanding contributes to the dynamic capabilities theory by illustrating how intelligence-driven decision-making fosters organizational adaptability.

**Bridging Strategic Intelligence with Innovation in Healthcare:** While strategic intelligence has been studied in business and competitive intelligence contexts, its role in organizational learning and innovation within healthcare has remained under-theorized. This study provides evidence that strategic intelligence is associated with both radical (exploratory) and incremental (exploitative) innovation, a pattern consistent with the resource-based view (RBV) of organizational performance.

**Providing a Theoretically Grounded Empirical Model:** This study develops and tests a structural equation model (SEM) linking strategic intelligence to organizational ambidexterity within a single county-level public healthcare network. Although the proposed framework provides empirical support for the hypothesized relationships, its applicability to other healthcare organizations and health systems should be evaluated through replication in diverse organizational and geographical contexts. By confirming the statistical significance of these relationships, this study advances strategic management and healthcare innovation theories.

In summary, our results are broadly consistent with, and extend, existing knowledge in this area, with three caveats appropriate to a single-site, cross-sectional study. First, the prominent association of partnering (β = 0.458) with OA is directionally consistent with accounts of collaborative decision-making in hospitals [24], although that source did not examine partnering as operationalized here, and the comparison should be read as thematic rather than as a direct replication. Second, the associations with exploitative innovation (H3) and exploratory innovation (H2) are consistent with Gibson and Birkinshaw's [26] ambidexterity framework applied to a healthcare setting. Third, the non-significant motivation path departs from the pattern reported in Maccoby's [19] original account of strategic intelligence; because this study did not measure crisis timing, workload, or intrinsic motivation directly, any explanation for this null result, including possible ceiling or saturation effects during high-demand periods, is speculative and is not asserted here as an explanation.

Systemic thinking (H5: β = 0.067, z = 1.036, *p* > 0.05) and motivation (H7: β = 0.058, z = 0.894, *p* > 0.05) were not statistically significant predictors of OA in this sample. This contrasts with some prior findings [12], [19]. Several explanations have been proposed in the literature for why particular SI dimensions might not relate to organizational outcomes in a given setting, for example role-based differences in exposure to interdepartmental dynamics [22], [23], information asymmetry between administrative and clinical staff [21], or the possibility that professional and clinical motivations operate somewhat independently of organizational strategic-alignment motivation as conceptualized by Maccoby [19]. We report these as candidate explanations drawn from the literature, not as conclusions supported by the present data, since this study did not directly measure organizational silos, information access, or the sources of employees' motivation, and the null results could equally reflect the sample size limitations discussed above (statistical power for these two paths was low; see Sensitivity analysis and Table 2) rather than a true absence of association. A further possible contributor is that the SI instrument [19] was developed and validated with managers in a different cultural and organizational context; its operationalization of systemic thinking and motivation may function differently for non-managerial healthcare staff completing a Persian translation, a measurement consideration distinct from the substantive explanations above. Taken together, whether the SI–OA association is context-dependent and role-contingent, as prior theory would suggest [7], cannot be established from these cross-sectional, single-site data and is proposed here as a direction for future research using multi-site samples and larger, adequately powered designs.

### 3.2 Practical implications

The organizational capabilities examined here, strategic intelligence and organizational ambidexterity, are discussed in the literature as potentially relevant to Sustainable Development Goal 3, because organizations that can respond to change and coordinate effectively are generally considered better positioned to support healthcare delivery. This study did not measure public health emergency response, clinical innovation implementation, multidisciplinary coordination, or healthcare delivery under changing conditions; the association reported here is between employees' perceptions of SI and OA, and any relevance to SDG 3 should be read as a potential, unverified implication rather than a demonstrated organizational improvement.

Whether strengthening strategic intelligence would improve healthcare quality, organizational preparedness, evidence-based decision-making, professional coordination, or emergency response is a plausible hypothesis raised by this literature; it was not tested in the present study and would require research designs that directly measure those outcomes.

Integrating strategic intelligence into healthcare leadership is proposed in the literature as a way to enhance adaptability and innovation; the present cross-sectional data cannot establish whether doing so would improve service delivery. The findings of this study provide significant practical insights for healthcare administrators, policymakers, and strategic decision-makers:

Enhancing decision-making in healthcare organizations: Given the complexity and volatility of healthcare environments, managers must rely on strategic intelligence to navigate regulatory shifts, technological advancements, and demographic changes [22], [23]. This study's findings are consistent with embedding foresight, vision, and partnering into healthcare decision-making processes, although the practical benefit of doing so was not directly tested.

Improving healthcare innovation through ambidexterity: Healthcare organizations are commonly described as needing to balance process efficiency (exploitation) with innovation (exploration). This research offers preliminary, association-based evidence relevant to that balance, though not a tested framework for crisis response or patient-care innovation specifically.

Building a culture of strategic foresight: The study emphasizes the importance of strategic foresight in preparing healthcare organizations for future uncertainties, such as pandemics, demographic shifts, and resource shortages. Whether incorporating predictive analytics or AI-driven tools would enhance resilience was not examined in this study and is noted here only as a direction raised by the broader literature.

Strengthening cross-departmental collaboration: The association between partnering and OA observed here is consistent with the idea that interdisciplinary collaboration matters for healthcare innovation, though this study measured perceptions of partnering rather than collaboration outcomes directly. Organizations should encourage knowledge-sharing initiatives and interdepartmental strategic planning.

Guiding policymakers in healthcare strategy development: These preliminary, single-site findings may be of interest to policymakers considering strategic-intelligence-related organizational development, though the present evidence does not by itself support specific policy recommendations without further, larger-scale research.

This study contributes preliminary empirical evidence on the association between strategic intelligence and organizational ambidexterity in a public healthcare network. It offers a starting point for future, larger, and multi-site research on these organizational capabilities in healthcare management.

### 3.3 Managerial discussions

The findings of this study offer significant practical insights for healthcare managers, policymakers, and organizational strategists. In an increasingly complex and dynamic healthcare environment, the ability to anticipate change, balance innovation with efficiency, and make informed strategic decisions is critical to organizational success. This research highlights the essential role of strategic intelligence in enhancing organizational ambidexterity, enabling healthcare institutions to simultaneously exploit existing competencies while exploring new opportunities for growth and innovation.

One of the key takeaways is the positive association between strategic intelligence and organizational ambidexterity observed in this sample, particularly through foresight, vision, and partnering. These associations are consistent with, but do not by themselves demonstrate, the value of long-term scenario planning, data-driven decision-making, and participatory leadership. The ability to forecast industry trends, regulatory shifts, and emerging healthcare needs is discussed in the literature as helping healthcare institutions remain proactive, which prior literature links to organizational conditions that may support patient safety, healthcare quality, continuity of care, and workforce responsiveness. None of these patient-related outcomes were measured in the present study, and organizational ambidexterity is discussed here as a potential, not a demonstrated, mechanism linking SI to health-system performance.

The study also emphasizes the importance of balancing exploratory and exploitative innovation. Many healthcare organizations struggle to innovate while maintaining efficiency, often prioritizing one at the expense of the other. The findings indicate that strategic intelligence is positively associated with both types of innovation in this sample, consistent with, but not proof of, the idea that it helps organizations pursue both at once. This is particularly relevant for healthcare systems facing digital transformation, increasing patient demands, and resource constraints.

Moreover, the positive association of partnering with organizational ambidexterity is consistent with the idea that involving employees in strategic decision-making matters for OA, although this study did not test leadership interventions directly. Encouraging collaborative leadership, cross-functional teamwork, and knowledge-sharing initiatives enhances employee engagement, fosters a culture of innovation, and improves adaptability. Healthcare managers should implement structured feedback mechanisms, continuous professional development programs, and interdisciplinary task forces to ensure that employees at all levels contribute to strategic initiatives.

From an operational perspective, leveraging advanced data analytics, artificial intelligence (AI), and digital health technologies are often discussed as potential supports for strategic intelligence capabilities, though evaluating their effect on healthcare service efficiency, cost, or patient-centered care was outside the scope of this study.

Finally, these preliminary findings may be of interest to healthcare policymakers considering strategic-intelligence-related organizational development. Given the single-site, cross-sectional design, we refrain from recommending specific policy measures and instead note this as a direction for future, more extensive research.

Strategic intelligence and organizational ambidexterity are organizational capabilities that were positively associated with each other in this healthcare network. Developing foresight and partnering, and supporting a balance between innovation and efficiency, are directions consistent with this study's findings, though further research is needed before drawing firm managerial conclusions about service delivery or organizational performance.

## 4 Conclusion

What this study demonstrates: In this public healthcare network, strategic intelligence was positively associated with organizational ambidexterity (H1,H2,H3), and among the SI dimensions, foresight, vision, and especially partnering were positively associated with OA, while systemic thinking and motivation were not statistically significant (H4–H8). These are the empirical findings of the study. What remains a proposed implication: whether these organizational associations translate into improved operational efficiency, innovation capacity, organizational resilience, preparedness, or responsiveness to changing healthcare needs was not tested here and should not be inferred from these results. This study did not directly measure patient-level health outcomes, healthcare quality, service accessibility, or other indicators of health-system performance; any statement connecting strategic intelligence to health-system performance in this article refers to a hypothesized pathway for future research, not to a result demonstrated by the present data.

From a public health perspective, the organizational capabilities examined here are conceptually related to Sustainable Development Goal 3 in the broader literature; this study's findings are offered as a preliminary empirical building block for that literature, not as evidence that investing in strategic intelligence constitutes a demonstrated health-system-strengthening strategy.

The findings of this study are grounded in and contribute to a growing body of literature that examines how organizations navigate uncertainty through adaptive innovation and ambidextrous decision-making. Studies on public-private partnerships in healthcare, digital health paradoxes, and VUCA-condition strategies [28], [29], [30], [31] have collectively demonstrated that organizational resilience in complex environments depends on balancing efficiency with innovation. Our empirical findings extend this literature by providing quantitative evidence that strategic intelligence serves as a key enabler of this balance in healthcare settings.

Specifically, our results align with the VUCA-condition literature by showing that healthcare organizations with stronger strategic intelligence capabilities—particularly in foresight, vision, and partnering—show higher perceived organizational ambidexterity, combining exploitation and exploration orientations. This adds cross-sectional quantitative evidence to a literature previously dominated by qualitative accounts, without establishing the mechanism by which these capabilities would translate into ambidexterity in practice.

Hence, the healthcare system has a complex and multilayered environment due to differences in the structures, resources, and objectives of the public and private sectors. Organizations in this field must both exploit existing resources and structures and pursue innovations such as telemedicine, big data analytics, and advanced therapies like gene therapy. The rapid advancement of technologies like artificial intelligence and digital treatments forces organizations to continuously adapt. Additionally, the diversity of stakeholders, including patients, physicians, insurance companies, and pharmaceutical firms, complicates decision-making. Crises such as the COVID-19 pandemic have made the future even more uncertain for these organizations. In such volatile and complex (VUCA) conditions, enhancing strategic intelligence is a key factor for balancing exploitation and innovation within organizations. Few studies have simultaneously examined these issues in the healthcare sector using structural models, which points to a need for further research and situates the contribution of the present, preliminary study.

The dimensions of strategic intelligence that showed a significant positive association with organizational ambidexterity in this sample were foresight, vision, and partnering. Employees who report a stronger ability to anticipate opportunities and threats in their environment also report higher OA in this cross-sectional sample; this association does not establish that foresight causes greater innovation or creativity. Managers and employees should develop their foresight as a special capability, based on a deep understanding of technological, demographic, legal, and lifestyle trends.

Foresight is a synthesis of many individuals' perspectives on recognizing the future, and employees' responsibility is to anticipate the challenges the organization will face in the future, which is a fundamental assumption of the innovation process. Vision is a key factor and a mental movement from the known to the unknown, enabling effective employees to create a compelling future for their community by integrating facts, desires, ideals, opportunities, and threats. Partnering, the fifth SI dimension, concerns the ability to build alliances and productive collaborative relationships; it is theorized to help organizations access external resources, promote synergies, and foster learning, which may support innovation. In this sample, partnering showed the strongest positive association with OA among the five SI dimensions.

In conclusion, strategic intelligence, as reported by employees in this healthcare network, was positively associated with organizational ambidexterity, which encompasses both exploratory and exploitative dimensions. The ability of health-service organizations to discover new opportunities and leverage existing competencies, leading to organizational ambidexterity, is associated in this study with foresight, a clear vision, and partnering among stakeholders within the organization.

Although the present research focuses on organizational behavior within a public healthcare network, its findings may be of interest to health-systems researchers because organizational capabilities are widely discussed as potential upstream contributors to healthcare delivery. Strategic intelligence and organizational ambidexterity may be worth examining in relation to healthcare organizations' response to emerging health challenges, implementation of organizational innovations, continuity of care, and adaptation to changing service demands. These organizational capabilities were not linked to patient-level or population-health outcomes in the present study, and any relevance to healthcare delivery or to Sustainable Development Goal 3 (Good Health and Well-being) is a hypothesis for future research rather than a finding of this study.

The findings should be interpreted within the organizational and institutional context of the Health and Treatment Network of Ferdows County, Iran. Because the study employed a single-site sample from one county-level public healthcare organization, the results should not be considered statistically representative of other healthcare organizations or healthcare systems. Instead, they provide context-specific empirical evidence that may be transferable to comparable healthcare settings with similar organizational characteristics. Future multi-site and cross-national studies are needed to determine the external validity and broader applicability of the proposed relationships across different healthcare systems.

Like any scientific investigation, this study has several limitations. First, the research was conducted within a single county-level public healthcare network in Ferdows, Iran. Consequently, the findings reflect the organizational characteristics of this specific healthcare setting and should not be interpreted as statistically representative of healthcare organizations or healthcare systems beyond the study context. Second, although the sample size was adequate for structural equation modeling, all participants were recruited from a single organizational setting, which limits the external validity and transferability of the findings. Future research should replicate the proposed model across multiple healthcare organizations, regions, and countries with different institutional, cultural, and healthcare system characteristics to evaluate the robustness of the observed relationships. In addition, longitudinal and multi-center studies would provide stronger evidence regarding the stability and consistency of these relationships over time and across diverse healthcare contexts. Finally, this study did not directly measure patient health outcomes, healthcare quality indicators, or other SDG 3 metrics. Therefore, the findings should be interpreted as evidence of organizational mechanisms that may support health system performance rather than as evidence of a direct relationship with patient or public health outcomes. Future studies should extend the proposed model by incorporating measurable healthcare outcomes, such as patient safety, patient satisfaction, quality of care, service accessibility, continuity of care, and other relevant SDG 3 indicators. Fifth, the sample size (*n* = 172) was modest relative to the complexity of Models 2 and 3, and statistical power for detecting smaller associations (including the non-significant systemic thinking and motivation paths) was limited; these results should be treated as preliminary pending replication in larger, multi-site samples (see the sample-size justification and Monte Carlo simulation reported above). Sixth, this study used causal-sounding constructs (strategic intelligence, ambidexterity) but a cross-sectional, single-time-point, self-report design; despite the procedural and statistical common-method-bias checks reported above, shared-method variance cannot be fully ruled out, and temporal precedence and causality cannot be established. Seventh, the Strategic Intelligence Questionnaire was originally validated with managers and was applied here to all healthcare employees; measurement invariance across occupational roles was not tested. Eighth, formal statistics for multivariate normality (e.g., Mardia's coefficient) and for the homogeneity-of-variance assumption underlying the job-unit ANOVA (e.g., Levene's test) were not computed for this analysis; both are recommended before the ANOVA and SEM results are relied upon further.

Subject to the limitations above, the results of this research are consistent with the Health and Treatment Network of Ferdows County supporting employees who propose innovative ideas and creating favorable conditions for staff growth and development. Additionally, managers should review and update existing methods and practices in light of external environmental changes. This requires providing a clear vision and foresight based on systemic thinking within the organization.

Future researchers are encouraged to replicate this model in other public healthcare networks and, where feasible, in other health-service settings, using larger and multi-site samples, and to extend it with directly measured healthcare-delivery outcomes. Other analytical approaches, such as multi-criteria decision-making techniques, could also help prioritize among the SI dimensions examined here.

## Generative AI disclosure statement

During the preparation of this manuscript, the authors used ChatGPT (OpenAI) to assist with language editing, improving clarity, enhancing academic writing style, and refining the organization of the manuscript. The authors critically reviewed, verified, and revised all AI-generated suggestions and accept full responsibility for the accuracy, originality, interpretation, and integrity of the final manuscript. No generative AI tool was used to generate, analyze, or interpret the research data, perform statistical analyses, or draw the scientific conclusions presented in this study.

During the preparation of this manuscript, the authors used AI to assist with language editing, improving clarity, enhancing academic writing style, and refining the organization of the manuscript. The authors critically reviewed, verified, and revised all AI-generated suggestions and accept full responsibility for the accuracy, originality, interpretation, and integrity of the final manuscript. No generative AI tool was used to generate, analyze, or interpret the research data, perform statistical analyses, or draw the scientific conclusions presented in this study.

## Patient involvement

No patient was involved.

## CRediT authorship contribution statement

**Mohammad Khalilzadeh:** Writing – review & editing, Visualization, Validation, Supervision, Software, Resources, Project administration, Methodology, Investigation, Formal analysis, Data curation. **Zahra Rajaei:** Writing – original draft, Visualization, Resources, Project administration, Methodology, Investigation, Formal analysis, Data curation, Conceptualization. **Sayyid Ali Banihashemi:** Visualization, Resources, Conceptualization.

## Consent for publication

Not applicable.

## Ethics approval and consent to participate

This study was approved by the [NAME OF INSTITUTIONAL RESEARCH ETHICS COMMITTEE/IRB], approval reference [APPROVAL NUMBER], approved on [APPROVAL DATE]. [AUTHORS: please insert the actual committee name, reference number, and approval date here before resubmission.] All procedures were performed in accordance with the Declaration of Helsinki. Prior to participation, employees were informed in writing about the purpose of the study, the voluntary nature of participation, and the anonymity and confidentiality of their responses, and informed consent was obtained from all participants before they completed the questionnaire. Because the study was conducted within participants' own employing organization, participation was explicitly described as voluntary and unrelated to employment evaluation or standing, and questionnaires were returned through a procedure that did not allow responses to be traced to individual employees, in an effort to reduce any perceived pressure to participate.

This article is the result of research conducted at Payame Noor University of South Khorasan province, approved by the Ethics Committee of Birjand University of Medical Sciences, under the number 1399.305 on 07/07/1399 (Persian calendar is June 27, 2020.) The committee waived the requirement for written informed consent, as the research involved minimal risk, voluntary participation of healthcare employees, and anonymized data collection. Verbal consent was obtained from all participants and recorded by the research team during questionnaire administration, in accordance with national guidelines for non-clinical studies. No patient data or vulnerable populations were involved. The authors express their deepest gratitude to all those who assisted in this research.

## Funding

The author(s) received no financial support for the research, authorship, and/or publication of this article.

## Declaration of competing interest

The authors declare that they have no competing interests.

## Data Availability

The dataset and AMOS output files analyzed in this study are available from the corresponding author upon reasonable request.
